# Decellularized Extracellular Matrix Scaffold Loaded with Regulatory T Cell-Conditioned Medium Induces M2 Macrophage Polarization

**DOI:** 10.34133/bmr.0196

**Published:** 2025-04-18

**Authors:** Hongjing Jiang, Xuheng Sun, Jiang Liu, Lijun Fang, Yuanfeng Liang, Jiahui Zhou, Yueheng Wu, Zhanyi Lin

**Affiliations:** ^1^ School of Medicine, South China University of Technology, 510006 Guangzhou, Guangdong, China.; ^2^ Guangdong Provincial People’s Hospital (Guangdong Academy of Medical Sciences), Southern Medical University, 510080 Guangzhou, Guangdong, China.; ^3^ Department of Geriatrics, Guangdong Provincial Geriatrics Institute, Guangdong Provincial People’s Hospital (Guangdong Academy of Medical Sciences), Southern Medical University, 510006 Guangzhou, Guangdong, China.; ^4^ Ji Hua Institute of Biomedical Engineering Technology, Ji Hua Laboratory, 528200 Foshan, Guangdong, China.

## Abstract

Biomaterials often induce local inflammatory responses following implantation. Scaffolds that cause continuous M1 polarization typically hinder tissue healing and regeneration. Regulating the transformation of macrophages to the M2 phenotype in the inflammatory environment is crucial. We propose that regulatory T cell-conditioned medium (T_reg_ CM) effectively promotes M2 polarization of macrophages induced by decellularized extracellular matrix (dECM) materials in inflammatory environments. In vitro results showed that in the presence of dECM, T_reg_ CM induces the polarization of RAW264.7 macrophages to M2 and inhibits M1 macrophage polarization under inflammatory conditions (lipopolysaccharide + IFN-γ). Additionally, dECM promotes the polarization of bone marrow-derived macrophages (BMDMs) to M2, while T_reg_ CM further promotes M2 polarization and inhibits M1 polarization in an inflammatory environment. These findings were confirmed by transcriptome sequencing. T_reg_ CM inhibited IκB kinase/NF-κB signaling and cellular responses to oxidative stress. In vivo subcutaneous transplantation showed an increase in M2 macrophages, a decrease in M1 macrophages, and an increased M2/M1 ratio in dECM materials loaded with T_reg_ CM. These results suggest that T_reg_ CM can create a pro-M2 polarized microenvironment for dECM, guiding immune responses toward favorable tissue regeneration. Ultimately, this research highlights the potential of T_reg_ CM as a therapeutic approach to modulate the immune response and improve the efficacy of regenerative biomaterials.

## Introduction

After implantation, ECM biomaterials interact with the host immune system, particularly macrophages [1]. Macrophages, an important cell type in the immune response, directly influence the biocompatibility of materials, as well as tissue repair and regeneration. Macrophages are typically divided into 2 polarization states: pro-inflammatory M1 and anti-inflammatory M2 [2]. M1 macrophages activate the acute inflammatory response and initiate immune rejection of foreign materials by secreting proinflammatory cytokines, such as tumor necrosis factor-α (TNF-α) and interleukin-1β (IL-1β), and producing reactive oxygen species (ROS) [2]. Commonly, this response is the initial reaction after implantation, and persistent M1 activation leads to local inflammation and tissue damage, potentially affecting material remodeling and regeneration [3]. Over time, macrophages undergo polarization, shifting toward M2 macrophages [3]. M2 macrophages have anti-inflammatory properties and secrete cytokines, such as IL-10 and transforming growth factor-β (TGF-β), which promote the healing and regeneration of injured tissues by reducing inflammation and promoting tissue repair and regeneration [3].

Recent research has revealed that, in addition to the generally described M2 phenotype, several subtypes have emerged: M2a, M2b, M2c, and M2d [4]. M2eff macrophages have also been reported [4]. While this classification improves phenotypic stratification, it likely oversimplifies macrophage phenotypes, as macrophages are thought to reside on a continuum of activation states, ranging from M1 to M2 [5]. Nonetheless, the M1/M2 nomenclature has proven beneficial as a predictor of in vivo ECM biomaterial remodeling outcomes [6] and as a prognostic marker for certain diseases [7]. We used the M1/M2 nomenclature, which is the standard for reporting results in primary sources [4]. Polarization from M1 to M2 plays a crucial role in tissue repair and regeneration. This process follows a clear timeline; usually, within the first few days after material implantation, M1 macrophages dominate, the local inflammatory response is gradually relieved, the number of M1 macrophages decreases, and M2 macrophages progressively increase, eventually dominating the immune response [3,8]. However, material properties, such as surface roughness, hydrophilicity, and biomolecular modification, can influence macrophage polarization direction. For example, macrophages on rough, mineralized collagen surfaces exhibit the M1 phenotype, while those on smoother surfaces exhibit the M2 phenotype [9]. Although hydrophilic/neutral surfaces inhibit macrophage adhesion, they increase cytokine production (IL-1, IL-6, IL-8, and IL-10) compared with hydrophobic and ionic surfaces [10]. Micro- and nano-patterned grooves influence macrophage elongation, and surface grooves do not affect inflammatory activation but guide macrophages toward an anti-inflammatory, pro-healing phenotype [11]. The macrophage phenotype was successfully modulated by injectable interferon-γ (IFN-γ) or IL-4, polarizing them into pro-inflammatory (M1) or pro-healing (M2a and M2c) phenotypes, respectively [12]. Alternatively, different tissue-derived decellularized extracellular matrix (dECM) has distinct matrisome protein compositions, and changes in these unique components can influence resident cells in a tissue-specific manner [13]. Therefore, designing and optimizing the properties of materials to guide the polarization of macrophages, from M1 to M2, is key for improving the repair function of biomaterials and promoting tissue regeneration.

Previous studies have explored the effects of different types of biomaterials on macrophage polarization [4,14,15]. Macrophages exposed to the small intestinal submucosa, urinary bladder matrix, brain ECM, esophageal ECM, and colon ECM predominantly express an M2-like phenotype, whereas exposure to dermal ECM results in a predominant M1-like phenotype [15]. Porcine brain decellularized matrix-based hydrogels promote macrophage polarization toward the M2 phenotype [16]. However, an acute inflammatory response is inevitable in the early stages following implantation. This response is mainly mediated by M1 macrophages, which promote immune rejection and tissue damage by secreting pro-inflammatory factors (e.g., TNF-α and IL-1β) and producing ROS [1,4,8]. Therefore, studying the effects of biomaterials on macrophage polarization in an ongoing inflammatory microenvironment is crucial. Effectively regulating macrophage polarization in a complex and changing inflammatory microenvironment, especially to promote polarization toward the M2 phenotype, which supports tissue repair, has become a major challenge in current research.

Regulatory T (T_regs_) cells are important immunoregulatory cells that maintain immune tolerance and suppress excessive immune responses by secreting immunosuppressive cytokines [17]. T_reg_ cell function depends on the expression of the transcription factor Foxp3 [17]. Foxp3 promotes the differentiation of T_reg_ cells and regulates their immunosuppressive effects. The main cytokines secreted by T_reg_ cells include IL-10, TGF-β, and IL-35, with IL-10 and TGF-β playing key roles in inhibiting inflammatory responses, promoting immune tolerance, and regulating tissue repair [17]. IL-10 reduces the inflammatory response by inhibiting the production of pro-inflammatory cytokines. TGF-β contributes to the establishment of immune tolerance and promotes the polarization of macrophages to M2 type, further promoting tissue repair and regeneration [18]. T_reg_-conditioned medium (T_reg_ CM) is a culture medium obtained by culturing T_reg_ cells and contains cytokines and other active molecules secreted by these cells. It has been reported that T_reg_ CM contains a variety of cytokines, including IL-10, IL-8, IL-6, IL-13, vascular endothelial growth factor (VEGF), IP-10, MCP-1, RANTES, and Osteopontin [19,20], with wide application potential in regulating immune responses, promoting tissue regeneration, and immunotherapy [21].

Previously, we observed that T_reg_ cells actively participate in constructive remodeling following the implantation of dECM materials [22]. Therefore, in this study, we combined in vitro and in vivo approaches to investigate the effect of T_reg_ CM on macrophage polarization in the presence of dECM within a persistent inflammatory microenvironment. We also loaded T_reg_ CM into dECM material to effectively regulate macrophage polarization toward the M2 phenotype after subcutaneous implantation. These findings provide new insights not only into improving the regenerative potential of biomaterials but also into the development of next-generation biomaterials.

## Materials and Methods

### Preparation of dECM material

We selected the Sprague–Dawley (SD) rat aorta for decellularization treatment to obtain dECM and observed its effect on mouse macrophages in vivo/in vitro studies. Artery-derived dECM has a denser structure and is more restrictive to host cell infiltration into the scaffold. Therefore, observing the regulatory effect of T_reg_ CM on macrophages in this heterogeneous dense dECM scaffold is more informative.

SD rats were purchased from the Guangdong Medical Laboratory Animal Center (Laboratory Animal Production License No. SCXK 2022-0002). The aorta (native) was removed from the SD rats and washed repeatedly with phosphate-buffered saline (PBS). The aortas of SD rats were rinsed for 18 h and decellularized with a mixture of zwitterionic detergent (8 mM CHAPS) and anionic detergent (1.8 mM SDS), as described previously [22]. After decellularization, the aortas were washed with PBS. The dECM was stored in sterile PBS at 4 °C. All procedures were performed at 37 °C with 5% carbon dioxide.

### H&E staining

Pre-decellularized (native) and post-decellularized (dECM) aortic samples were fixed in 4% paraformaldehyde (PFA) for 24 h. Samples were then soaked in 50% alcohol, 70% alcohol, 80% alcohol, 95% alcohol, and 100% alcohol for complete dehydration. After tissue transparency was completed, the tissue was immersed in wax replacing xylene with paraffin. After embedding, 5-μm-thick paraffin sections were prepared for hematoxylin and eosin (H&E) staining.

### DNA quantification

The materials from the native and dECM groups were freeze-dried under vacuum. The samples were digested overnight in papain solution (125 μg/ml papain, 5 mM cysteine, and 5 mM EDTA). The DNA concentration was quantified using the PicoGreen Quantitative Assay kit (PicoGreen, Invitrogen, USA) according to the manufacturer's instructions.

### Scanning electron microscopy

Samples from the native and dECM groups were fixed in 2.5% glutaraldehyde at 4 °C overnight. The next day, the fixed liquid was removed by double steam washing and gradient dehydration was performed using a series of ethanol solutions. The samples were placed on aluminum stubs, air-dried, and then sputter-coated with gold (Leica, Germany). A Verios 5 UC SEM (Thermo Fisher Scientific, USA) was used for imaging.

### Live/dead cell assay

The dECM material was placed in complete medium at a ratio of 2.5 ml per square centimeter and incubated with 5% CO_2_ at 37 °C for 24 h. Complete medium without dECM under the same conditions was used as a control. Human fibroblasts (5 × 10^5^ cells/ml, CTCC-180-HUM, Meisen CTCC, China) were cultured in a mixture of the extract and complete culture medium (1:1, dECM:complete medium) in 2 groups, dECM and control, for 48 h. Cell viability was assessed using a live/dead cell assay kit (Beyotime, China). Images of live and dead cells were captured using a fluorescence microscope (Olympus, Tokyo, Japan). Cell viability [(%) = (number of calcein-AM^+^ cells)/(number of calcein-AM^+^ cells + number of PI^+^ cells) × 100] was calculated.

### Preparation of the T_reg_ CM

The EasySep Mouse CD25 Treg Pos Sel Kit (18782; StemCell, Canada) was used to isolate T_reg_ cells from the spleens of 8-week-old SPF C57BL/6 mice. T_reg_ cells were cultured in RPMI 1640 medium (HyClone, USA) supplemented with CD28 (1 μg/ml, 102115, BioLegend, USA), IL-2 (30 ng/ml, PeproTech, USA), and 10% fetal bovine serum (FBS; Corning, USA). Prior to cell plating, culture plates were coated with CD3 antibodies (10 μg/ml, 100339, BioLegend, USA). Following 3 d of culture, CD3 and CD28 were eliminated, and 10% FBS and IL-2 (30 ng/ml, PeproTech, USA) were used to cultivate T_reg_ cells. After collecting the conditioned medium, cells were centrifuged for 10 min at 3,000 rpm. The supernatant was collected, passed through a 0.22-μm membrane filter, and stored at −80 °C.

### Polarization of RAW264.7

In an inflammatory microenvironment, RAW264.7, mouse macrophages, and solubilized dECM material were cultured in vitro with or without T_reg_ CM. Solubilized dECM material was prepared by grinding the dECM material into a powder. The ground powder was freeze-dried as previously reported [15]. Pepsin (Sigma-Aldrich) was used to dissolve the ECM solution containing 10 mg/ml dECM. A 1:10 volume of NaOH (0.1 N) and a 1:9 volume of 10× PBS were added for neutralization.

The mouse macrophage cell line RAW264.7 was purchased from MeilunBio (PWE-MU004, MeilunBio, China) and cultured constantly at 37 °C with 5% CO_2_ using high-glucose Dulbecco’s modified Eagle’s medium (DMEM) medium supplemented with 10% FBS and 1% penicillin–streptomycin. Single-cell suspensions were obtained from cells in the logarithmic growth stage and inoculated into a 6-well cell culture plate at a concentration of 2 × 10^5^ cells/well. The cells were cultured overnight, and different intervention treatments were given after cellular adherence: (a) M0 group, untreated RAW264.7 macrophages in complete medium; (b) inflammation treatment + dECM group in complete medium + 100 ng/ml lipopolysaccharide (LPS) (Sigma-Aldrich, USA), 20 ng/ml IFN-γ (PeproTech, USA), and 200 μg/ml dECM; (c) inflammation treatment + dECM + T_reg_ CM group in complete medium and T_reg_ CM (1:1 ratio) + 100 ng/ml LPS, 20 ng/ml IFN-γ, and 200 μg/ml dECM. The cells in each group were treated for 24 h, and follow-up tests were performed.

### Immunocytochemistry

The cells were fixed in 4% PFA for 30 min at room temperature (Sigma-Aldrich, USA). The cells were permeabilized with 0.25% Triton X-100 for 20 min, followed by 3 washes with PBS. The cells were blocked with 3% bovine serum albumin (Sigma-Aldrich, USA) for 30 min. Cells were incubated with primary antibodies 4 °C overnight. The following antibodies were used: anti-iNOS (ab178945; Abcam, UK), CD206 (AB64693; Abcam, UK), and CD68 (sc-20060; Santa Cruz Biotechnology). The concentration of each antibody was determined according to the manufacturer’s instructions. After 3 PBS washes, DyLight 594 goat anti-rabbit immunoglobulin G (IgG) (1:500, A23420, Abbkine, China), DyLight 488 goat anti-rabbit IgG (1:500, A23220, Abbkine, China), and DyLight 594 goat anti-mouse IgG (1:500, A23410, Abbkine, China) secondary antibodies were added and samples were incubated for 1 h at room temperature.

Nuclei were stained with 4′,6-diamidino-2-phenylindole (DAPI). Three independent samples were taken for each experiment, at minimum, and 3 to 5 random visual fields were analyzed for each sample. All images were collected using an Olympus IX-81 inverted fluorescent microscope (Olympus IX-81, Olympus Corporation, Japan) with the same parameters (laser power, gain, and exposure time) maintained across all experimental conditions. ImageJ software was used to measure and quantify fluorescence intensity, which was normalized to that of the control group.

### Western blotting

The RAW264.7 cells were collected and washed 3 times with pre-cooled PBS. Protease inhibitors (Thermo Fisher Scientific) and radioimmunoprecipitation assay (RIPA) lysis buffer (Beyotime Biotechnology, China) were added to the samples for 30 min on ice. After centrifuging the samples for 15 min at 4 °C and 12,000 rpm, supernatants were collected. The bicinchoninic acid assay (BCA)(Thermo Fisher Scientific) was used to measure the amount of protein in each sample. Protein samples were separated by 10% sodium dodecyl sulfate–polyacrylamide gel electrophoresis, electro-transferred to nitrocellulose membranes, and blocked with milk. inducible nitric oxide synthase (iNOS) (1:1,000, ab178945, Abcam, UK) and β-actin (1:10,000, 66009, Proteintech, USA) were then added. Goat anti-rabbit conjugated horseradish peroxidase (1:20,000; Abbkine, China) was used as secondary antibody. Following the manufacturer’s instructions, the proteins were detected using the enhanced chemiluminescence method (Biosharp, China). All protein band densities were quantified with ImageJ software, using β-actin as internal control. All quantifications were normalized to the control group.

### RNA extraction and real-time RT-PCR of mRNA

Total RNA was extracted using TRIzol reagent (Invitrogen, Carlsbad, CA, USA) according to the manufacturer’s instructions. The PrimeScript RT reagent kit (TaKaRa, Japan) was used to reverse transcribe 1,000 ng of total RNA into cDNA. The Hieff qPCR SYBR Green Master Mix (YEASEN, China) and qTOWER3G Real-Time RT-PCR System (Jena, Germany) were used for real-time reverse transcription polymerase chain reaction (RT-PCR) analysis. Using 2^−∆∆Ct^ method, relative mRNA expression levels were calculated by RT-quantitative PCR (RT-qPCR) with β-actin as the internal control. Expression values were normalized to the control group. Table S1 lists the primer sequences used for the real-time RT-PCR.

### Polarization of bone marrow-derived macrophages

The femur and tibia were sterilely harvested from 8-week-old SPF C57BL/6 mice. The bone was sliced from both ends and rinsed with RPMI 1640 medium to extract the bone marrow. The bone marrow was washed 3 times changing the bone marrow cavity to a white color. RBC lysis buffer (Solarbio, China) was used to lyse the red blood cells after the cell suspension was filtered with a 70-μm cell strainer. Prior to culture, the cells were resuspended in RPMI 1640 medium supplemented with macrophage colony-stimulating factor (M-CSF) (20 ng/ml, PeproTech, USA) and 10% FBS at a concentration of 2 × 10^6^ cells/ml. After 7 d of culturing, replacing the medium every 2 d, M0 macrophages (non-polarized BMDMs) were obtained. M0 macrophages were split into different groups and underwent different treatments: (a) inflammatory treatment (LPS + IFN-γ): RPMI 1640 medium with 100 ng/ml LPS and 20 ng/ml IFN-γ; (b) inflammation + dECM treatment: RPMI 1640 medium with 100 ng/ml LPS, 20 ng/ml IFN-γ, and 200 μg/ml dECM; (c) inflammation + dECM + T_reg_ CM treatment: RPMI 1640 medium mixed with T_reg_ CM (1:1 ratio) with 100 ng/ml LPS, 20 ng/ml IFN-γ, and 200 μg/ml dECM. Cells in each group were treated under the above conditions for 48 h before subsequent analyses.

### Transcript sequencing and data analysis

The BMDMs were analyzed using transcriptome sequencing. TRIzol reagent (Invitrogen, USA) was used to isolate and purify total RNA. RNA integrity was evaluated using the Agilent Technologies Bioanalyzer 2100. Following quality inspection, the library was sequenced using an Illumina Novaseq 6000. RNA transcripts were assembled, their expression levels were determined using StringTie software, and the sequencing data were compared with the reference genome using Hisat software. Differential expression analysis was performed using edgeR. The findings were graphically displayed using R software, including principal components analysis, volcano maps, and heatmaps of the differentially expressed genes. Gene Ontology (GO) and Kyoto Encyclopedia of Genes and Genomes (KEGG) pathway enrichments were used for additional functional analyses.

### dECM freeze-dried adsorbed T_reg_ CM

The dECM was placed into blocks of 0.5 cm length, 0.5 cm width, and 0.1 cm height. The blocks were placed into a lyophilizer to ensure complete dryness of the dECM. The lyophilized dECM was then placed into T_reg_ CM and adsorbed overnight at 4 °C. The dECM adsorbed in RPMI 1640 medium supplemented with 30 ng/ml IL-2 and 10% FBS was used as the control group under the same conditions.

### Cytokine release experiment

To determine the time course of cytokine release after loading T_reg_ CM with dECM material, we used TGF-β and IL-10 as assay markers. The dECM materials of the control group and T_reg_ CM group were placed in PBS and mixed on a shaker at 37 °C. PBS was collected at each time point and stored at −20 °C. The levels of released TGF-β and IL-10 were measured by the TGF-β enzyme-linked immunosorbent assay (ELISA) kit (EH0012, HUABIO, China) and IL-10 ELISA kit (EM0005, HUABIO, China), respectively, following the manufacturer’s instructions.

### Subcutaneous implantation

All experimental animals were purchased from Guangdong Medical Laboratory Animal Center (Laboratory Animal Production License No. SCXK 2022-0002). Eight-week-old specific pathogen-free (SPF) C57BL/6 mice were used to establish the subcutaneous implantation model. All mouse experiments were performed in accordance with the experimental protocol approved by the Ethics Committee of Guangdong Provincial People's Hospital (approval ID: KY2023-201-01). All animals were kept under SPF conditions (24 °C, 12-h light/12-h dark cycle, 50% humidity) with access to pellet food and tap water ad libitum. The mice were handled in accordance with the Guide for the Care and Use of Laboratory Animals. Aseptic procedures were performed during all experiments.

After the mice were anesthetized with an intraperitoneal injection of sodium pentobarbital (45 mg/kg), their back hair was removed using an electric razor. Two incisions were made 0.5 cm to the left and right sides of the back midline and were used as surgical sites. The incisions were bluntly separated to form small cystic cavities. The dECM from the control and T_reg_ CM groups was placed in the cyst cavity, fitting within the back tissue of the mice. The dECM and T_reg_ CM groups were implanted into the upper left and lower right. The wound was closed using 5-0 surgical sutures. Wound healing, diet, and body weight was monitored daily. Five mice were used for each group (*n* = 5). Seven days after implantation, the mice were sacrificed under anesthesia, and the grafts and their surrounding tissues were isolated. Post-isolation, samples were immediately placed in 4% PFA solution, stored at 4 °C, and fixed for 24 h before subsequent experiments were performed.

### Tissue immunofluorescence staining

For histological analysis, the dECM samples were progressively dehydrated using low-to high-gradient concentrations of alcohol. Samples were immersed in wax and cut into 5-μm-thick sections. The paraffin sections were deparaffinized in water and subjected to antigen retrieval at high temperature and pressure. Sections were blocked with 10% donkey serum (AntGene, China) and subsequently subjected to antibody incubation as previously described [22]. The following antibodies were used: CD68 [1:400,97778, Cell Signaling Technology (CST), USA], CD86 (1:400,19589, CST, USA), and CD206 (1:100, 24595, CST, USA). After incubation with secondary antibodies, DAPI working solution was added dropwise, and the slides were sealed for microscopic examination. The entire dECM material was scanned using a 3DHistech (Pannoramic MIDI, Hungary). DAPI^+^, CD68^+^DAPI^+^, CD68^+^CD206^+^DAPI^+^, and CD68^+^CD86^+^DAPI^+^ cells were counted within the entirety of the sample. The data were presented as the proportion of positive cells. The M2/M1 ratio was calculated by dividing the number of M2 macrophages by the number of M1 macrophages.

### Statistical analysis

GraphPad Prism (version 9.0, USA) was used for analysis, charting, and statistics. All experimental results represent at least 3 independent replicates. Mean ± SEM was used to express all numerical values. The Student's *t* test with a 2-tailed distribution was used to compare 2 experimental groups. Tukey's post hoc test was used to complete a one-way analysis of variance (ANOVA) for multiple experimental group comparisons. The degree of significance is indicated by the following asterisks: *P ≤ 0.05, **P ≤ 0.01, ***P ≤ 0.001, ****P ≤ 0.0001. All *P* values < 0.05 were regarded as statistically significant. *P* values greater than 0.05, denoted by “ns”, indicated no statistical significance.

## Results

### Preparation and characterization of the dECM material

**Fig. 1.**
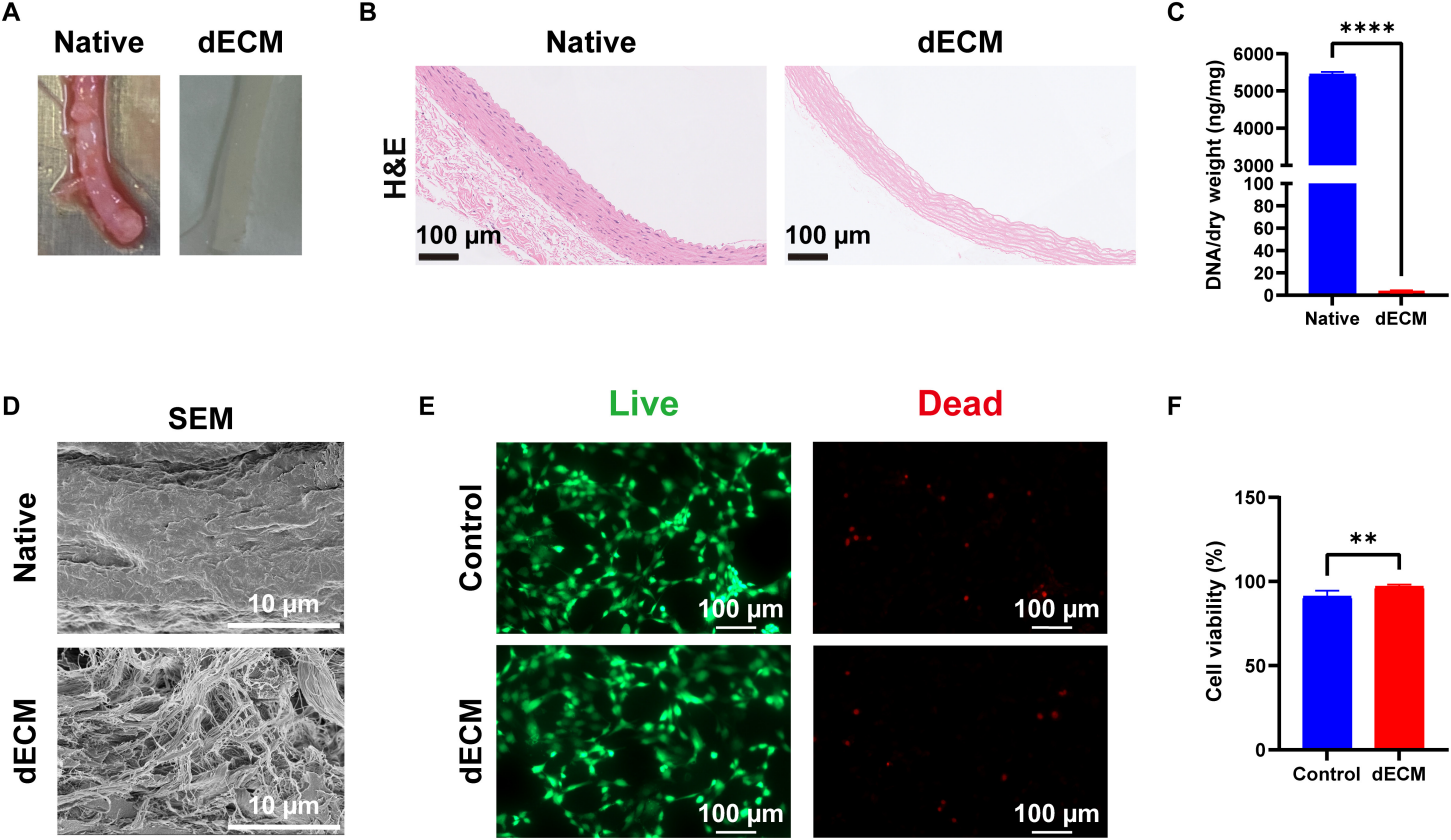
Characterization of the dECM material. (A) Representative images of native and dECM starting materials and (B) H&E-stained sections. (C) DNA concentrations in native and dECM materials were detected using PicoGreen. (D) Scanning electron microscopy images of the native and dECM materials. (E) Representative images of fibroblasts stained with propidium iodide (PI) (red) and calcein-AM (green) in an extract-contact cytotoxicity experiment. (F) Statistical analysis of cell viability based on calcein-AM/PI staining. A complete medium without dECM under the same conditions was used as the control group during extraction. In each experiment, *n* ≥ 3 biological replicates. Bars indicate the mean ± SEM. **P ≤ 0.01, ****P ≤ 0.0001.

After removing the aorta from SD rats, we performed decellularization using SDS + CHAPS (Fig. 1A). After decellularization, native rat arteries (native) contained a large number of nuclei, whereas dECM had a negligible amount of nuclei remaining (Fig. 1B). PicoGreen test showed that double-stranded DNA (dsDNA) content in the dECM was less than 50 ng/mg dry weight (Fig. 1C), which met the current decellularization standards. Scanning electron microscopy analysis confirmed that dECM retained a 3-dimensional ECM scaffold (Fig. 1D). The viability of fibroblasts after exposure to dECM extract was assessed using live/dead staining. The results showed that cell viability was 91.09% and 96.96% in the control and dECM groups, respectively. The difference between means (dECM − Control) ± SEM was 5.873 ± 1.641, with a statistically significant difference (*P* ≤ 0.01). These results suggest that the dECM marginally enhances fibroblast viability (Fig. 1E and F).

### In the inflammatory microenvironment, T_reg_ CM further promotes M2 polarization of RAW264.7 macrophages

**Fig. 2.**
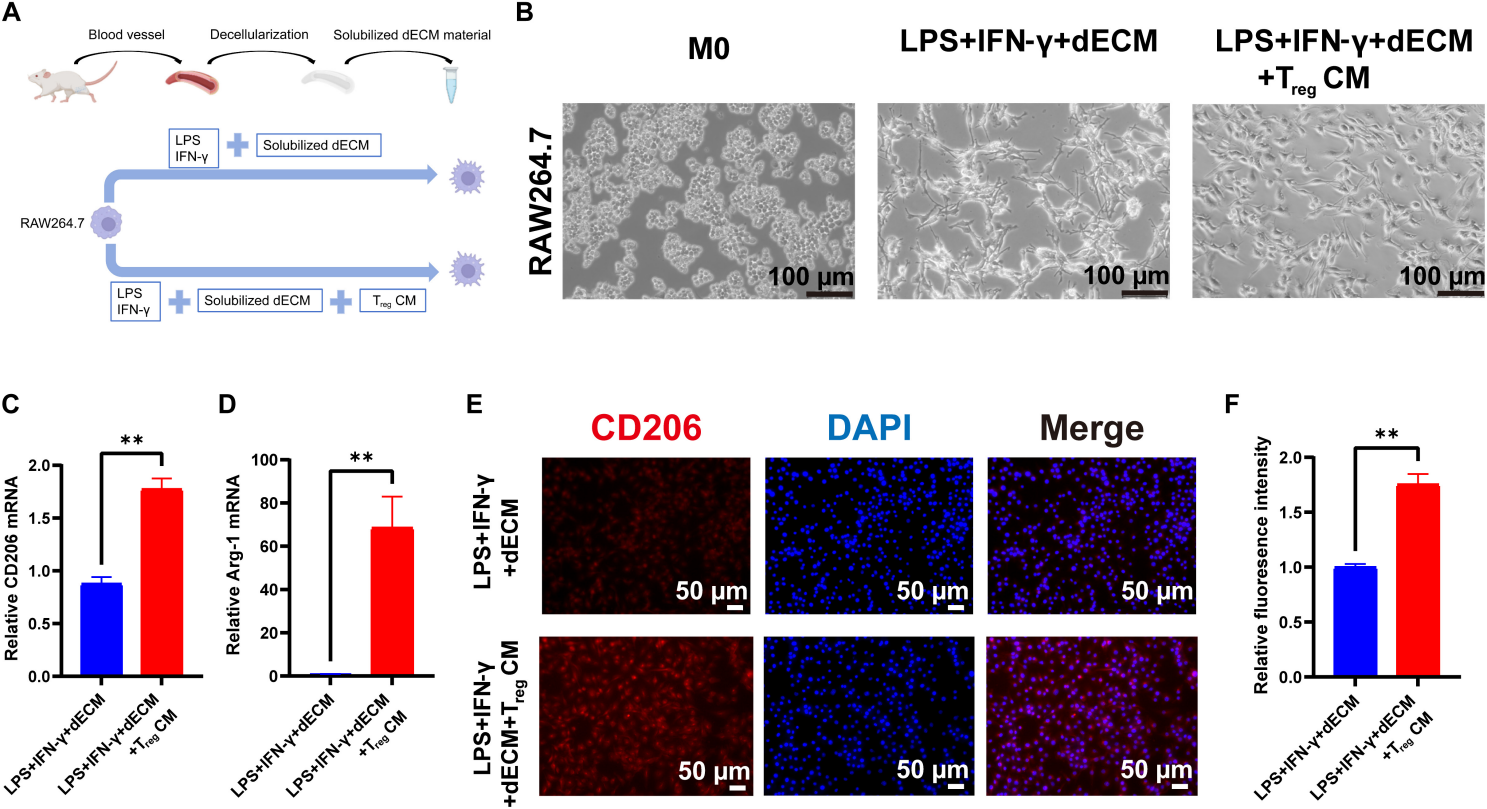
T_reg_ CM promotes M2 polarization of RAW264.7 macrophages cocultured with dECM material in an inflammatory microenvironment. (A) Experimental flow for RAW264.7 in vitro polarization. (B) Light microscopic diagram of RAW264.7 cells in each group. (C and D) RT-qPCR was used to measure the relative mRNA expression of *CD206* (C) and *Arg-1* (D) in M2 macrophages after T_reg_ CM addition. (E and F) Representative immunofluorescence images (E) and quantitative analysis (F) of CD206 expression. Each experiment had an *n* ≥ 3 biological replicates. Bars indicate the mean ± SEM. **P ≤ 0.01.

Previously, we found that increased recruitment of T_reg_ cells in vivo can promote the constructive remodeling of dECM materials in vivo, suggesting correlation with cytokine secretion by T_reg_ cells [22]. We used LPS + IFN-γ to induce macrophages to M1 polarization, and during this process, we cocultured them with dECM materials. Simultaneously, we compared the polarization of macrophages in the presence or absence of T_reg_ CM, exploring the regenerative capacity of T_reg_ CM to create an M2 microenvironment for dECM (Fig. 2A). RAW264.7 macrophages (M0, untreated RAW264.7 macrophages) were round or oval with a standard morphology, as expected (Fig. 2B). In the inflammatory microenvironment, RAW264.7 macrophages cocultured with dECM (LPS + IFN-γ + dECM) displayed differentiated cellular morphology, displaying the typical morphology of M1-type macrophages (Fig. 2B). However, in the LPS + IFN-γ + dECM + T_reg_ CM group, the macrophages showed typical morphology of elongated M2-type cells (Fig. 2B). The RT-qPCR results showed that the addition of T_reg_ CM promotes the mRNA expression of *CD206* and *Arg1* (M2 polarization markers) in macrophages, and their expression was significantly higher compared with macrophages in the LPS + IFN-γ + dECM group (Fig. 2C and D). This was further confirmed by immunofluorescence staining for CD206 (Fig. 2E). Fluorescence intensity for marker CD206 was increased in the LPS + IFN-γ + dECM + T_reg_ CM group (1.76 ± 0.10 relative to the LPS + IFN-γ + dECM group, *P* < 0.01) (Fig. 2F).

### In the inflammatory microenvironment, T_reg_ CM further inhibited M1 polarization of RAW264.7 macrophages

**Fig. 3.**
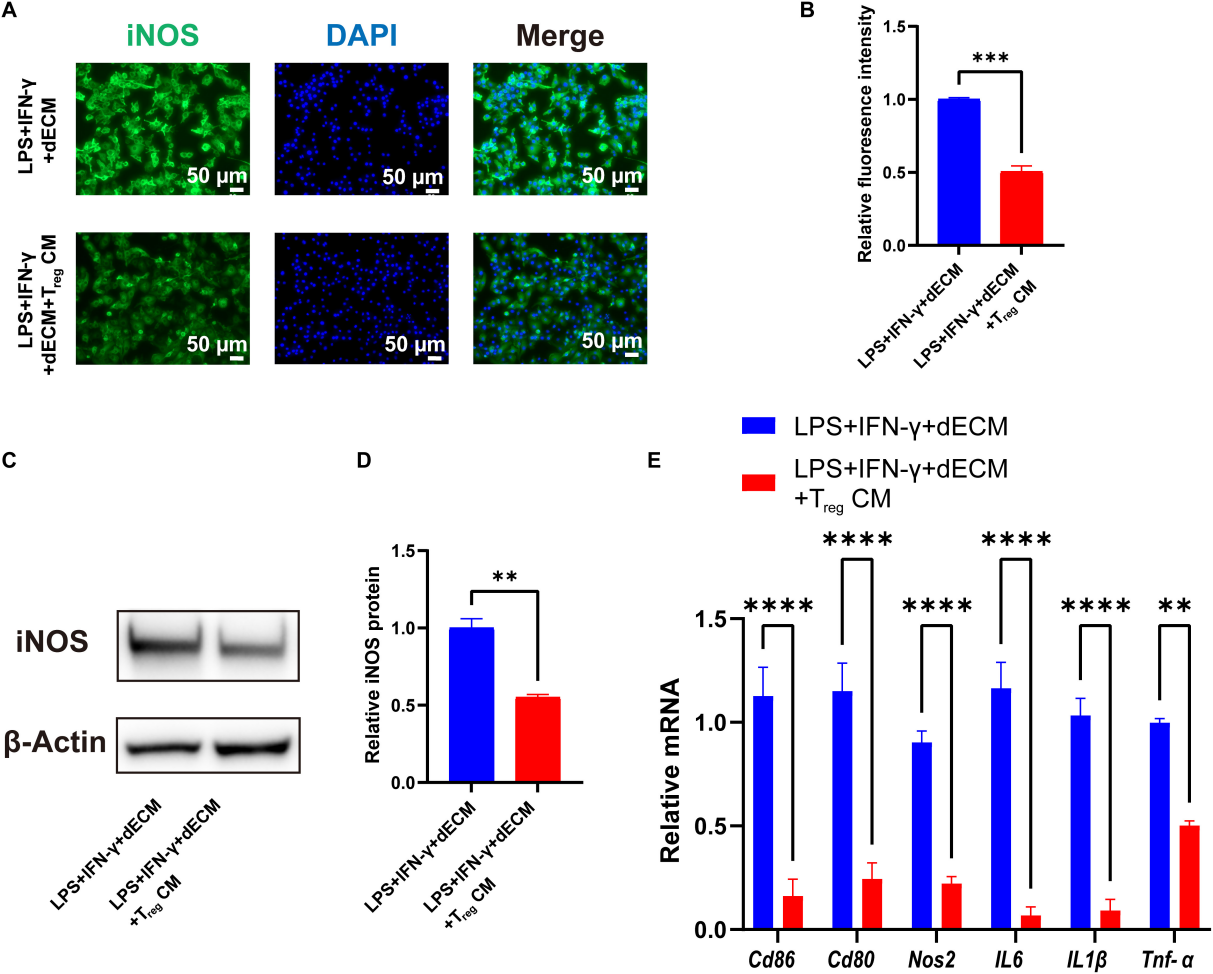
T_reg_ CM inhibited M1 polarization of RAW264.7 macrophages cocultured with dECM material in an inflammatory microenvironment. (A and B) Representative immunofluorescence images of iNOS (A) and the corresponding quantitative analysis (B). (C and D) Western blot of iNOS protein expression (C) and the corresponding quantitative analysis (D). (E) RT-qPCR was performed to detect relative mRNA expression levels associated with M1 macrophages after T_reg_ CM addition. Each experiment had a sample size of *n* ≥ 3 biological replicates. Bars indicate the mean ± SEM. **P ≤ 0.01, ***P ≤ 0.001, ****P ≤ 0.0001.

We examined the expression of M1-related markers. Immunofluorescence showed decreased expression of iNOS in the LPS + IFN-γ + dECM + T_reg_ CM group (Fig. 3A and B). Consistently, Western blot detection of corresponding iNOS protein was decreased (0.55 ± 0.02 relative to the LPS + IFN-γ + dECM group, *P* < 0.01) (Fig. 3C and D). The expressions of *CD86*, *CD80*, *Nos2*, *IL6*, *IL1β*, and *Tnf-α* were significantly reduced in the LPS + IFN-γ + dECM + T_reg_ CM group (Fig. 3E). These experiments suggest that T_reg_ CM, coupled with dECM, creates a regenerative M2 microenvironment. Within the inflammatory microenvironment, the presence of T_reg_ CM effectively inhibited the polarization of macrophages to the M1 type, inducing macrophage polarization into the M2 phenotype.

### dECM material regulates gene expression in BMDM macrophages

**Fig. 4.**
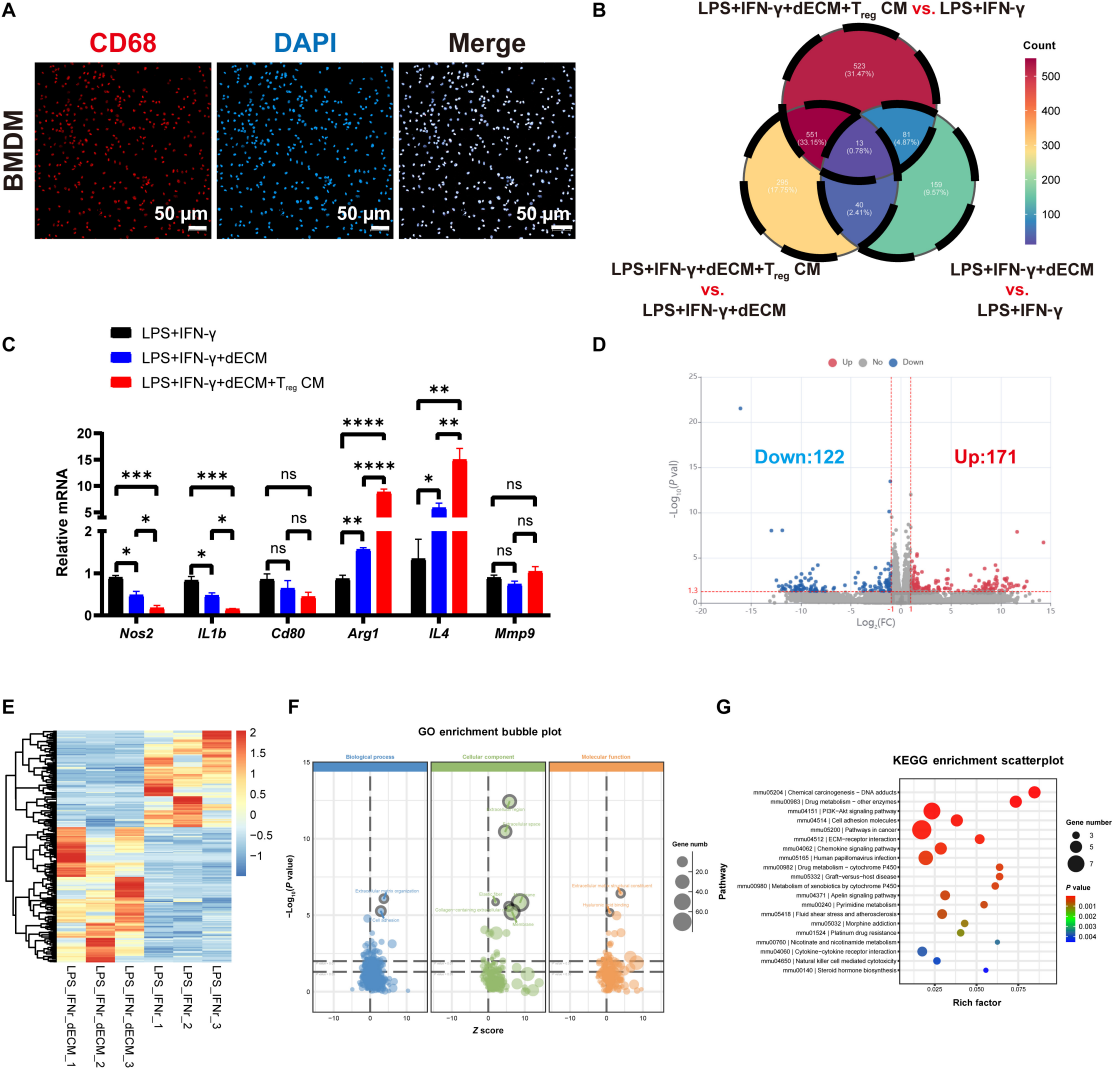
Gene expression regulation in BMDM by dECM within an inflammatory microenvironment. (A) Representative CD68 immunofluorescence staining in BMDMs. (B) Venn diagram of differentially expressed genes within each group. (C) RT-qPCR results detecting the relative mRNA expression within each group. (D to G) Volcano plot (D), heatmap (E), GO terms (F), and KEGG terms (G) of the differentially expressed genes within LPS + IFN-γ and LPS + IFN-γ + dECM groups. In each experiment, *n* ≥ 3 biological replicates. Bars indicate the mean ± SEM. ns = not significant, *P ≤ 0.05, **P ≤ 0.01, ***P ≤ 0.001, ****P ≤ 0.0001.

To fully elucidate the potential T_reg_ CM’s influence on macrophages, we isolated bone marrow-derived macrophages (BMDMs) from living tissues. The use of BMDMs aimed to exclude the potential influence of immortalized genetic modification to influence macrophage polarization. Immunofluorescence analysis of primary macrophages isolated, induced, and cultured from the bone marrow showed that these cells expressed the macrophage marker CD68 (Fig. 4A). LPS + IFN-γ induced macrophage polarization toward M1. Subsequently, we performed transcriptome sequencing of BMDM from each culturing condition group. The addition of both dECM and T_reg_ CM regulated gene expression in BMDMs (Fig. 4B).

We selected genes with previously defined associations with macrophage polarization and performed mRNA validation using RT-qPCR, validating the expression patterns of these genes under these conditions (Fig. 4C). Compared with the LPS + IFN-γ group, the addition of dECM material significantly decreased the expression of M1 polarization-related genes, *Nos2* and *IL1b*. Furthermore, the presence of T_reg_ CM enhanced this effect and further decreased the expression of M1 polarization-related genes, *Nos2* and *IL1b* (Fig. 4C). The expression of *Cd80* showed a similar, but statistically insignificant, trend (Fig. 4C). Similarly, compared with the LPS + IFN-γ group, the addition of dECM material significantly enhanced the expression of M2 polarity-related genes, *Arg1* and *IL4*. Furthermore, the presence of T_reg_ CM further increased *Arg1* and *IL4* expression levels (Fig. 4C). This suggests that in an inflammatory microenvironment, dECM promotes the polarization of BMDM toward M2 and inhibits the polarization toward M1. Consistently, T_reg_ CM enhances this effect, fostering a more pro-regenerative microenvironment for dECM materials. Additionally, there was no significant difference in the expression of *mmp9* among the groups (Fig. 4C).

The volcano map shows significant changes in transcripts between LPS + IFN-γ and LPS + IFN-γ + dECM groups (Fig. 4D), identifying 293 differentially expressed genes. Of these, 171 and 122 were up-regulated and down-regulated in LPS + IFN-γ + dECM cells, respectively (Fig. 4E). GO enrichment analysis revealed that the differentially expressed genes within the samples were predominantly involved in ECM organization, cell adhesion, extracellular region, extracellular space, collagen-containing extracellular matrix, elastic fiber, extracellular matrix structural constituents, and hyaluronic acid binding (Fig. 4F). KEGG enrichment analysis revealed significant enrichment of differentially expressed genes in multiple pathways, including the PI3K–Akt signaling pathway, cell adhesion molecules (CAMs), ECM–receptor interaction, chemokine signaling pathway, apelin signaling pathway, and cytokine–cytokine receptor interaction (Fig. 4G).

### T_reg_ CM modulates BMDM transcriptional phenotype, enhancing M2-related genes and reducing M1-related genes

**Fig. 5.**
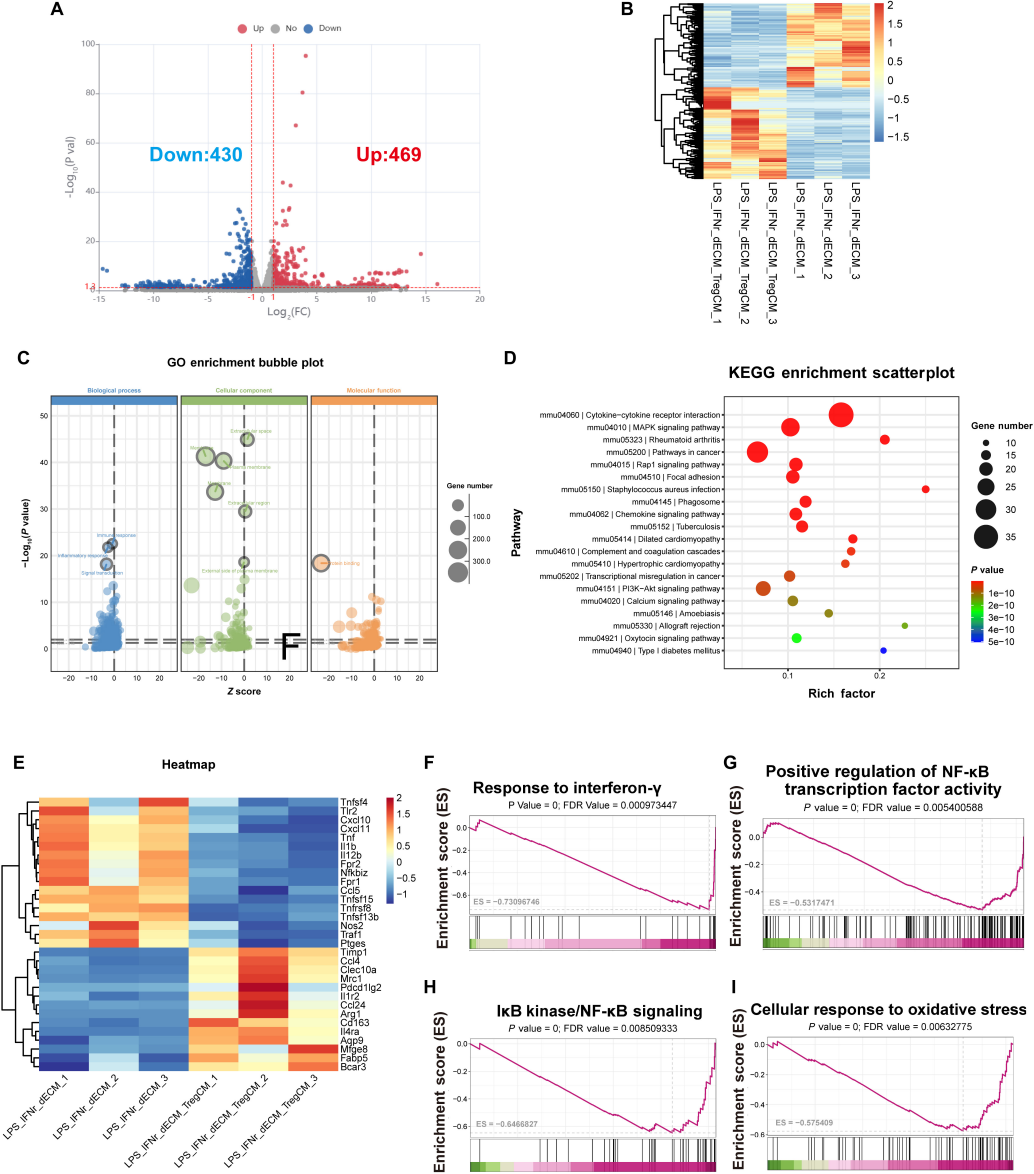
The transcriptional phenotype of BMDMs shows increased expression of M2-related genes and decreased the expression of M1-related genes after coculture with T_reg_ CM. (A to D) Volcano plot (A), heatmap (B), GO terms (C), and KEGG terms (D) of differentially expressed genes between LPS + IFN-γ + dECM and LPS + IFN-γ + dECM + T_reg_ CM groups. (E) Heatmaps of differentially expressed genes associated with macrophage polarization and inflammation in LPS + IFN-γ + dECM and LPS + IFN-γ + dECM + T_reg_ CM groups. (F to I) GSEA path diagram.

To further explore the effect of T_reg_ CM addition on BMDMs, we performed transcriptome analysis from LPS + IFN-γ + dECM and LPS + IFN-γ + dECM + T_reg_ CM groups. The volcano plot shows significant changes in transcripts between LPS + IFN-γ + dECM and LPS + IFN-γ + dECM + T_reg_ CM groups (Fig. 5A). In total, 899 differentially expressed genes were identified. Of these, 469 and 430 were up-regulated and down-regulated, respectively, in the cells of the LPS + IFN-γ + dECM + T_reg_ CM group (Fig. 5B). GO functional enrichment analysis of the differentially expressed genes suggested a predominant involvement in the immune response, inflammatory response, signal transduction, inflammatory response, extracellular space, plasma membrane, the external side of the plasma membrane, membrane, and protein binding (Fig. 5C). KEGG enrichment analysis revealed that the differentially expressed genes were significantly enriched in several pathways, including the cytokine–cytokine receptor interaction, mitogen-activated protein kinase (MAPK) signaling pathway, Rap1 signaling pathway, focal adhesion, phagosome, chemokine signaling pathway, complement and coagulation cascades, and PI3K–Akt signaling pathway (Fig. 5D).

Differentially expressed genes obtained by transcriptome sequencing were further analyzed to identify genes related to BMDM polarization and inflammation, before and after the addition of T_reg_ CM, displayed within heatmaps (Fig. 5E). Comparative literature reported macrophage polarization-related genes [2]. Within our dataset, we find the M1 macrophage-related genes (Tnf, Il1b, nos2, Il12b, and Nfkbiz) to have significantly lower expression, and the M2 macrophage-related genes (mrc1, arg1, cd163, clec10a, and pdcd1lg2) to have significantly higher expression (Fig. 5E). This suggests the validation of the polarization patterns of RAW264.7 cells cocultured with dECM, confirming that the addition of T_reg_ CM effectively inhibits the polarization of macrophages toward M1 and promotes the polarization toward M2.

The enrichment pathway results of transcriptome Gene Set Enrichment Analysis (GSEA) analysis showed that the response to IFN-γ pathway was significantly inhibited in the LPS + IFN-γ + dECM + T_reg_ CM group compared with BMDM macrophages treated with dECM alone (Fig. 5F). This suggests that macrophages in the T_reg_ CM group had a reduced response to IFN-γ. IFN-γ is an important driver of M1 macrophage polarization, which promotes the secretion of inflammatory factors by activating the nuclear factor-κB (NF-κB) pathway [23]. Inhibition of this pathway in the T_reg_ CM group indicated that the activation of M1-type macrophages was attenuated, resulting in a lower proportion of M1-type macrophages. Second, positive regulation of NF-κB transcription factor activity and IκB kinase/NF-κB signaling were inhibited (Fig. 5G and H). NF-κB is a key transcription factor for M1 macrophage polarization and is responsible for regulating the secretion of inflammatory factors [24]. The IκB kinase/NF-κB signaling pathway regulates the activation of M1 macrophages and inflammatory response by promoting the activation of NF-κB [24]. In the T_reg_ CM group, the inhibition of NF-κB signaling suggests that the M1-associated inflammatory response was reduced, further driving the tendency of macrophages to polarize toward the M2 type. Additionally, we found that the cellular response to oxidative stress pathway in the LPS + IFN-γ + dECM + T_reg_ CM group was also inhibited (Fig. 5I). M2 macrophages generally exhibit a lower oxidative stress response and tend to reduce ROS production to promote anti-inflammatory effects and tissue repair [25]. In contrast, M1-type macrophages produce large amounts of ROS during activation, leading to increased oxidative stress [25]. Thus, suppression of the oxidative stress response further indicated that the number of M1-type macrophages was reduced and that of M2-type cells was increased in the T_reg_ CM group. Furthermore, we found that the addition of T_reg_ CM inhibited the TNF-mediated signaling pathway (Fig. S1) and that TNF plays a key role in the activation of M1-type macrophages. Inhibition of the TNF signaling pathway can reduce the pro-inflammatory effect of M1 macrophages and help macrophages polarize into M2 macrophages [23]. Collectively, the inhibition of these pathways contributes to the reduced activation of M1-type macrophages and enhances the anti-inflammatory and repair functions of M2-type macrophages.

### Post-subcutaneous implantation promotes the polarization of M2 macrophages

**Fig. 6.**
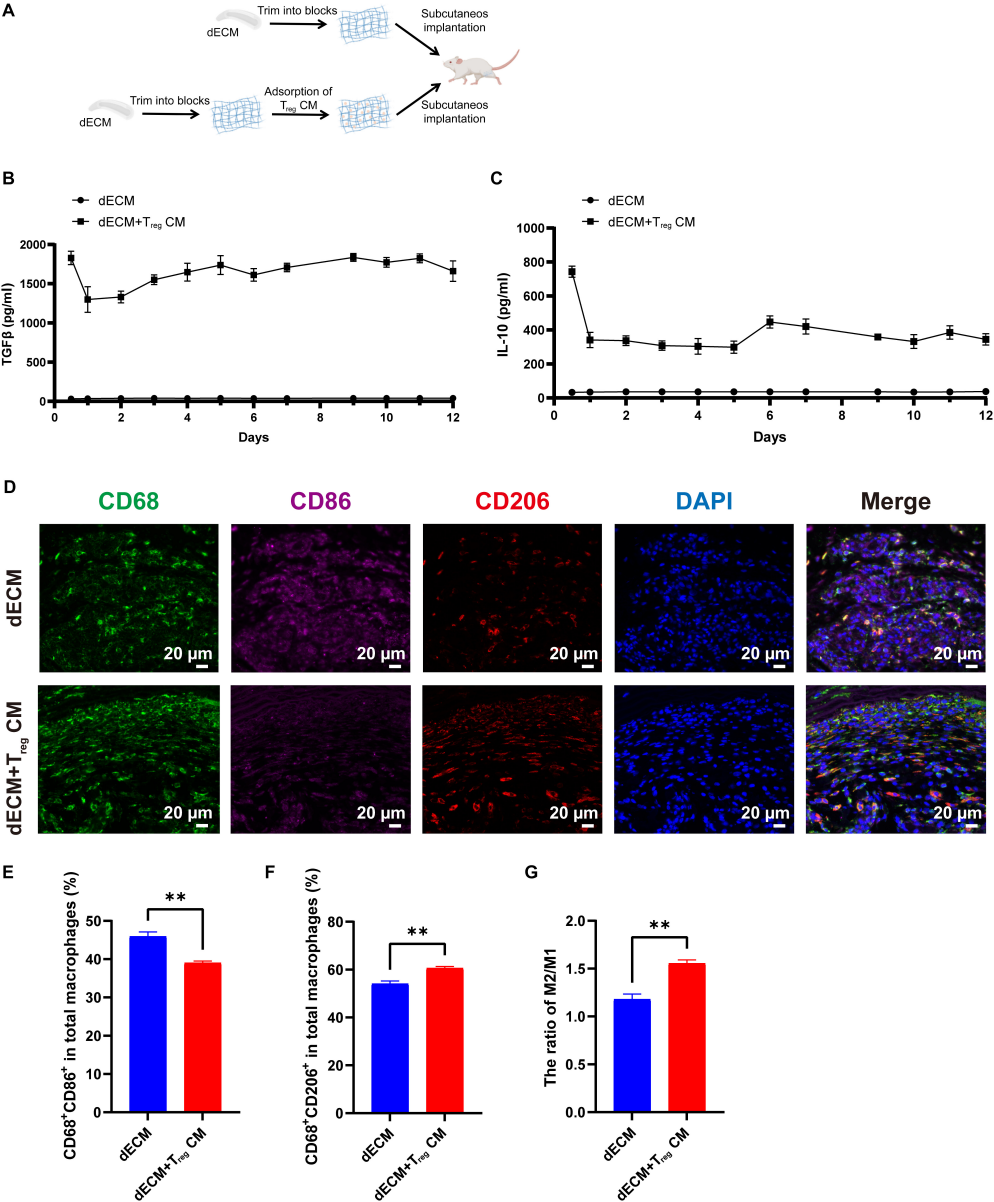
Post-subcutaneous implantation, the dECM scaffold material loaded with T_reg_ CM promoted the polarization of M2 macrophages. (A) Subcutaneous implantation protocol. (B and C) The time process of releasing TGF-β (B) and IL-10 (C) from dECM material after loading T_reg_ CM was detected by ELISA. (D to G) Representative immunofluorescence images of macrophage polarization markers inside dECM materials (D) and quantitative analysis of M1 macrophages (E), M2 macrophages (F), and M2/M1 ratio (G). In each experiment, *n* ≥ 3 biological replicates. Bars indicate the mean ± SEM. **P ≤ 0.01.

Macrophages are the main immune cells involved in inflammatory responses; therefore, it is important to study their polarization in vivo. dECM scaffolds rapidly cause an acute inflammatory response after implantation, recruiting a large number of macrophages to the graft area. In the early stages of implantation (0 to 7 d), the macrophages were predominantly of the M1 phenotype. We hypothesized that T_reg_ CM could still promote macrophage polarization toward the pro-regenerative M2 phenotype within this early inflammatory microenvironment in vivo. T_reg_ CM was adsorbed onto dECM using a lyophilized adsorption method. The combined material was then implanted subcutaneously into the backs of mice to observe the effect of T_reg_ CM on the polarization of macrophages in vivo (Fig. 6A). TGF-β and IL-10 are important components of T_reg_ CM. Using ELISA, we detected the release of TGF-β and IL-10 from dECM after adsorption of T_reg_ CM. The results showed that the dECM group released minimal amounts of TGF-β and IL-10, while the dECM + T_reg_ CM group continuously released TGF-β and IL-10 for up to 12 d (Fig. 6B and C). Tissue immunofluorescence was performed 7 d after subcutaneous implantation (Fig. 6D). In the dECM group, M1 macrophages were abundantly expressed (purple, CD86), whereas in the T_reg_ CM group, the proportion of M1 macrophages significantly decreased (Fig. 6E). Furthermore, in the dECM group, minimal M2 macrophage infiltration was observed; conversely, in the T_reg_ CM group, a significant increase in M2 macrophage infiltration was observed (red, CD206) (Fig. 6F). The M2/M1 ratio was higher in the dECM + T_reg_ CM group than in the dECM group (Fig. 6G). Lastly, we detected infiltrated CD45^+^ cells inside the dECM, resulting in a slight decrease of the number of infiltrated CD45^+^ cells inside the dECM + T_reg_ CM group, albeit this difference was not statistically significant (Fig. S2).

Collectively, the above results show that at the beginning of dECM material implantation, activation of the body’s immune response and M1 macrophage expression increases. The addition of T_reg_ CM causes a polarization shift, promoting M1 macrophages to decrease and M2 macrophages to increase proportionately. This suggests that T_reg_ CM improved the local immune microenvironment under inflammatory conditions, regulated the polarization of macrophages to the M2 phenotype, inhibited local adverse inflammatory reactions, promoted the transition to the repair stage, and regulated tissue repair.

## Discussion

Biomaterials play an important role in tissue replacement and repair. Biomaterials can trigger foreign body reactions following implantation. However, excessive inflammation can lead to the aggregation, growth, and proliferation of fibroblasts and macrophages on the surface of biomaterials, and the formation of fibrous encapsulation, leading to the failure of biomaterial implantation [26]. In the early stages of the inflammatory response, macrophages appear at the site of inflammation and persist for a long time. Through the classical activation pathway, macrophages polarize into the M1 phenotype, secreting high levels of pro-inflammatory cytokines and inflammatory mediators. Macrophages polarize into the M2 phenotype through alternative pathways, permitting clearance of the inflammatory response and exhibiting anti-inflammatory properties. Microenvironment changes influence the polarization phenotypes of macrophages, transforming fluidly into each other, resulting in dynamic participation within immune responses. Therefore, regulating macrophage polarization by changing the immune microenvironment is important for promoting early stage tissue repair [27]. Previous studies indicate that proper regulation of the immune microenvironment is conducive to regeneration [28]. At present, it has been reported that the ECM can promote macrophages to the M2 phenotype in vitro [29]. Furthermore, studies on macrophage-regulated biomaterials observe the polarization of dECM materials (modified/unchanged) after direct or indirect contact with macrophages [30]. However, macrophage responses to inflammation in the early stage influence the state and degree of macrophage differentiation in later stages, directly affecting the remodeling and regeneration of dECM materials [6,31]. Therefore, this study used LPS and IFN-γ to simulate the inflammatory environment to observe whether T_reg_ CM could affect the polarization of macrophages after contact with dECM materials. This study aimed to clarify the specific role of T_reg_ CM in the process of macrophage polarization (especially in the early stage of inflammation). These changes were detected after subcutaneous transplantation of T_reg_ CM mixed with dECM materials.

After preparing the dECM, live/dead cell staining assessed the viability of fibroblasts exposed to the dECM extracts. The results showed that the dECM extract minimally increased fibroblast viability. Other studies have shown similar results, where dECM did not markedly affect fibroblast survival [32], but promoted cell proliferation and migration [33,34]. This is partly due to the ability of dECM to retain its native constituents, including structural components and biochemical signaling molecules, such as growth factors [35,36]. A collagen scaffold functionalized into a soluble form, dECM, can provide a favorable microenvironment with higher levels of cytokines and growth factors, faster than a collagen scaffold functionalized in a particle form [37]. Skin-derived extracellular matrix (S-dECM) was used as a bioink for tissue-engineered skin. S-dECM retained the major ECM components of the skin, as well as favorable growth factors and cytokines. The viability of fibroblasts in the bioink was maintained at >90% by day 7 [38].

Next, we used the mouse macrophage cell line RAW264.7 as a culture model and stimulated the cells with LPS and IFN-γ to investigate the polarization of macrophages in the presence of T_reg_ CM and dECM. We analyzed the cell morphology, gene expression, and protein expression. Macrophages exhibit a high degree of plasticity and respond dynamically to multiple factors in the local microenvironment, thereby inducing complex, mixed phenotypes. First, we found noticeable morphological changes in RAW264.7 cells. The results showed that the addition of T_reg_ CM promoted the transformation of RAW264.7 into M2 macrophages, with high expression of M2 cell surface marker (CD206) and decreased expression of M1 cell marker (iNOS). Changes in the gene and protein levels of macrophages suggest changes in their functional status. T_reg_ CM group macrophages highly expressed anti-inflammatory genes (*CD206* and *Arg1*) and decreased inflammation-related genes (*CD86*, *CD80*, *Nos2*, *IL6*, *IL1β*, and *TNF-α*). These cytokines are known to polarize macrophages to M1 and participate in inflammatory responses.

The RAW264.7 cell line was selected due to its advantages of high homogeneity, easy availability, and easy culture of cell lines. Considering that the cell line is immortalized after transformation, its phenotype and function may differ from those of the primary cells. Thus, murine BMDM and the murine macrophage cell line J774 were used to study *Mycobacterium tuberculosis* infection. Marked differences were observed in the response patterns and degrees of response between the 2 cell types [39]. To clarify the effect of T_reg_ CM cocultured with the dECM scaffold on the polarization of primary macrophages, we isolated and cultured BMDM, and comprehensively evaluated their gene transcription levels using RNA sequencing technology. Of note, we did not include the gene expression data of M0 macrophages herein; however, studies have reported that M0 macrophages do not express *iNos*, *CD206*, and *Arg 1* [40].

By comparing the transcriptome data of BMDM under inflammatory conditions with and without exposure to dECM materials, we found that dECM materials significantly altered gene expression in BMDM. Most dECM biomaterials promote M2 macrophage polarization [15]. Immune regulation typically depends on the composition and structure of a scaffold; however, the exact mechanism has not been fully elucidated [41,42]. Studies have shown that this effect can be mediated by matrix-bound microvesicles embedded in the ECM [43]. This study revealed similar results. GO enrichment analysis showed that the differentially expressed genes in the samples were predominantly involved in the organization and function of extracellular matrix, including regulation of collagen, elastic fibers, and hyaluronic acid. This suggests that changes in matrix remodeling, cell adhesion, and signal transmission occur under these conditions. The abnormal regulation of these functions may be closely related to tissue repair, inflammatory responses, or changes in the tumor microenvironment. KEGG enrichment analysis showed that these pathways covered metabolism, signal transduction, and immune regulation. Notably, among the signaling pathways closely related to immune regulation, the PI3K–Akt signaling pathway was significantly enriched. This pathway is strongly involved in the regulation of immune cell survival, proliferation, and function. Additionally, it is closely related to the activation and effector functions of a variety of immune cells [44]. This result suggests that activation of the immune system may be further amplified through the PI3K–Akt pathway, thereby forming a multilevel immune regulatory network.

By comparing inflammation after contact with dECM material under BMDM and inflammatory conditions and T_reg_ CM and dECM material after contact with BMDM transcriptome data, we found that the expression of M1 macrophage-related genes (*Tnf*, *IL1b*, *Nos2*, *IL12b*, and *Nfkbiz*) was significantly lower and that of M2 macrophage-related genes (*Mrc1*, *Arg1*, *CD163*, C*lec10a*, and *Pdcd1lg2*) was significantly higher (Fig. 5E). We performed GO functional enrichment analysis of the differentially expressed genes obtained from transcriptome sequencing. The results showed that differentially expressed genes play an important role in immune and inflammatory responses and may affect cell recognition and response to pathogens through the regulation of signal transduction and transmembrane processes. The significant enrichment in the outer space of the plasma membrane and extracellular space further supports the hypothesis that these genes are involved in the perception and transmission of external signals. Combined with the enrichment of protein-binding functions, we speculated that these genes may play a central role in the activation of immune cells, cytokine secretion, and inflammatory mediator release. KEGG enrichment results showed that the differentially expressed genes were significantly enriched in immune-related pathways, such as cytokine interactions, MAPK signaling, and chemokine signaling pathways. This suggests that these genes participate in the host anti-infective immunity and inflammatory responses by regulating immune cell activation, inflammatory factor release, and signal transduction. The addition of T_reg_ CM caused a decrease in some members of the matrix metalloproteinase (MMP) family. However, the expression of some members of the tissue inhibitor of the matrix metalloproteinase (TIMP) family increased (Fig. S3). Previously, we showed that reduced T_reg_ recruitment by dECM scaffolds leads to the rapid degradation of collagen and elastic fibers in the scaffold [22]. We found that *Timp1* was the most significantly up-regulated gene after the addition of T_reg_ CM (Fig. S4), which may explain why T_reg_ cells prevent the rapid degradation of dECM scaffolds.

Specifically, in the inflammatory microenvironment, the polarization pattern of T_reg_ CM to macrophages was consistent with the results of the cell lines. The polarization level of M1 was significantly decreased, while that of M2 was significantly increased. The results obtained from primary cells further suggest that T_reg_ CM could play an important role in the regeneration process of dECM materials by influencing the polarization of early M1 macrophages, verifying the advantages of T_reg_ CM in reducing the inflammatory response of macrophages and improving the pro-regeneration microenvironment for dECM.

Furthermore, we observed the role of T_reg_ CM in the complex microenvironment in vivo. Macrophages begin to interact with biomaterials at an early stage of implantation, and their interaction determines the fate of biomaterials and their effect on tissue regeneration [45]. The adsorption of T_reg_ CM by dECM continuously releases TGF-β and IL-10 in vitro for up to 12 d. TGF-β and IL-10 are important components of T_reg_ CM, and it has been established that TGF-β promotes macrophage polarization to M2 type [46]. IL-10 is an important anti-inflammatory cytokine that governs M2 polarization through signal transducer and activator of transcription 3 (STAT3) activation via the IL-10 receptor (IL-10R) [47]. Additionally, IL-10 inhibits the polarization of M1 macrophages by inhibiting the activation of signaling pathways such as NF-κB and STAT1 [24,47]. Macrophage polarization is influenced by the microenvironment, leading to the hypothesis that cytokines in T_reg_ CM (e.g., TGF-β and IL-10) contribute to the suppression of M1 macrophages and the increase of M2 polarization.

After dECM implantation in vivo, macrophage infiltration and polarization were analyzed using immunological indicators. When the M2/M1 ratio around the scaffold is high, the probability of human tissue regeneration increases [48]. Our results showed that T_reg_ CM significantly decreased the proportion of M1 macrophages, increased the infiltration and proportion of M2 macrophages, and increased the M2/M1 ratio. Scaffolds that cause sustained M1 polarization are usually not conducive to tissue healing and regeneration, and a timely increase in the proportion of macrophages expressing M2 polarization indicators at the implantation site often indicates a good prognosis. dECM scaffolds that promote constructive functional remodeling show a higher proportion of M2 macrophages than scaffolds that promote scar tissue formation and encapsulation [6]. Therefore, our in vivo study demonstrated that T_reg_ CM could provide a pro-regenerative microenvironment for the dECM to induce the transformation of macrophages into M2 pro-regenerative and pro-repair phenotypes.

In future studies, we will extend the time point of the in vivo study to further observe the extent of maintaining the pro-regenerative effect of T_reg_ CM on the dECM materials. Additionally, patients with chronic diseases, such as diabetes, have a higher degree of inflammation in their bodies than those without systemic chronic diseases [49]. Previous studies have shown that chronic inflammation adversely affected biomaterial regeneration in vivo [50]. Therefore, future experiments can focus on the effects of T_reg_ CM in chronic inflammatory environments. Biomaterials carrying cytokines that regulate the immune microenvironment, such as T_reg_ CM, could suppress local inflammation and promote biomaterial regeneration in chronic disease models.

In summary, a dECM was prepared and the effects of various immunological components on the polarization of macrophages were observed. The addition of T_reg_ CM changed the morphology of RAW264.7 macrophages after dECM exposure under inflammatory conditions, promoted the polarization of RAW264.7 macrophages to M2, and inhibited the polarization of RAW264.7 macrophages to M1. Primary BMDM further confirmed that the addition of dECM material significantly decreased the expression of M1 polarization-related genes and increased the expression of M2 polarization-related genes. Furthermore, the presence of T_reg_ CM enhanced this effect on BMDM. The inflammatory microenvironment of subcutaneous transplantation was constructed in vivo and demonstrated that T_reg_ CM mixed with dECM could construct a pro-M2 polarization microenvironment, promoting macrophage infiltration into the material for M2 polarization. An increase in the M2/M1 ratio promoted an inflammatory environment guided by the promotion of immune pathways that are favorable to tissue regeneration. These findings can be translated into the design of biomaterials using T_reg_ CM, ultimately improving the remodeling and regeneration of dECM scaffolds.

## Data Availability

Data will be made available on request.
