# Supplementary material for: Decellularized Extracellular Matrix Scaffold Loaded with Regulatory T Cell-Conditioned Medium Induces M2 Macrophage Polarization
Source: Biomater Res. 2025 Apr 18;29:0196. doi: 10.34133/bmr.0196 (PMC12006722; doi:10.34133/bmr.0196)
Supplement: Supplementary 1 — Figs. S1 to S4 Table S1 [file bmr.0196.f1.docx]

SUPPLEMENTARY MATERIALS


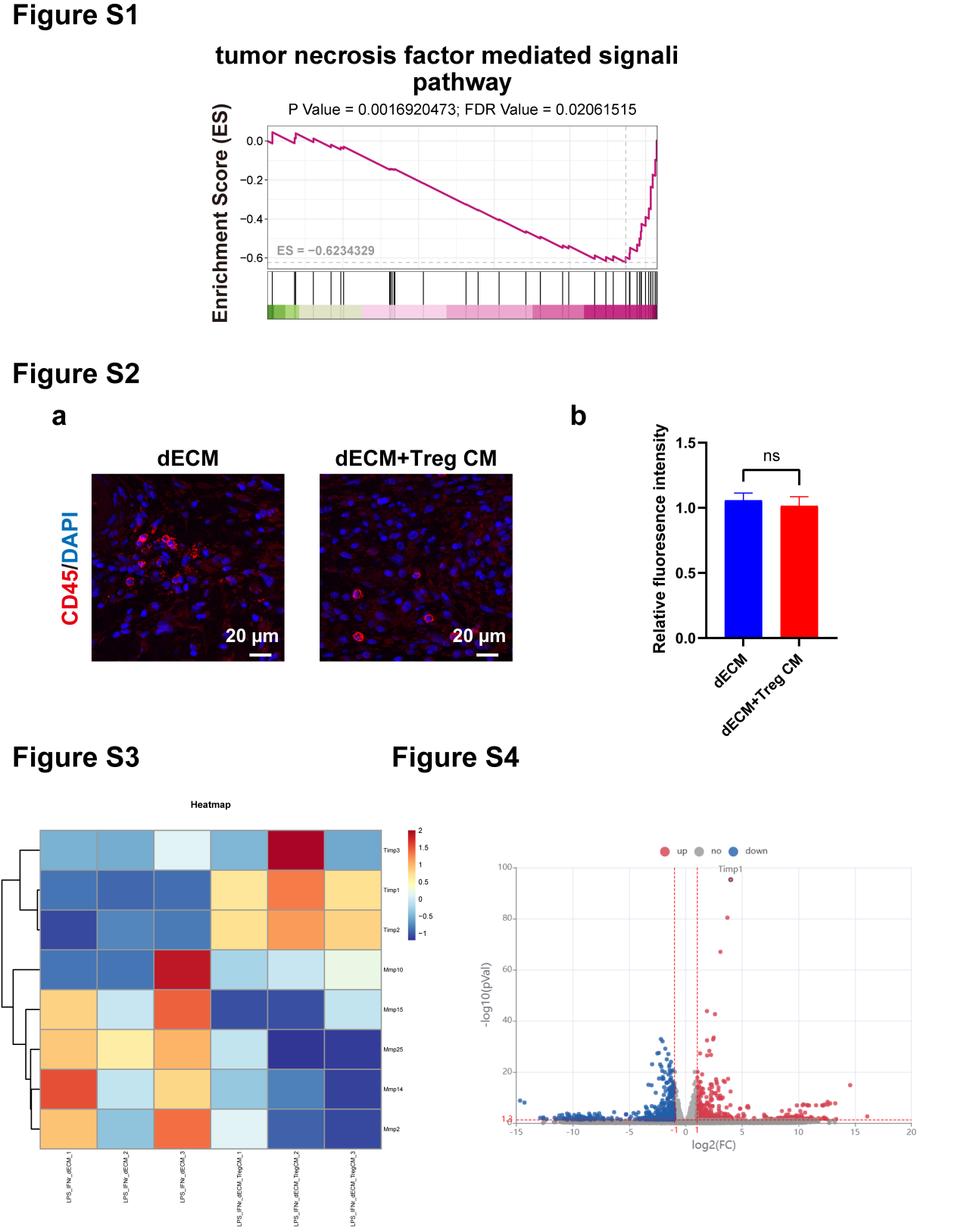


Figure S1. GSEA path diagram.

Figure S2. (A,B) Representative immunofluorescence images of CD45+ cell inside dECM materials (A) and quantitative analysis (B).

Figure S3. Heat maps of differentially expressed genes within Timp and Mmp families in LPS+IFN-γ+dECM and LPS+IFN-γ+dECM+Treg CM groups.

Figure S4. The volcano map of differential genes showed that Timp1 was the most significantly upregulated gene after the addition of Treg CM.

Table S1. Primer sequences used for RT-qPCR.

| Gene | Forward | Reverse |
| --- | --- | --- |
| *CD86* | TGTTTCCGTGGAGACGCAAG | TTGAGCCTTTGTAAATGGGCA |
| *CD80* | ACCCCCAACATAACTGAGTCT | TTCCAACCAAGAGAAGCGAGG |
| *Nos2* | GGAGTGACGGCAAACATGACT | TCGATGCACAACTGGGTGAAC |
| *IL6* | TAGTCCTTCCTACCCCAATTTCC | TTGGTCCTTAGCCACTCCTTC |
| *IL1β* | TTTGAAGTTGACGGACCCCA | TGTTGATGTGCTGCTGCGAG |
| *TNFα* | AGAAGTTCCCAAATGGCCTC | CCACTTGGTGGTTTGCTACG |
| *CD206* | CTCTGTTCAGCTATTGGACGC | CGGAATTTCTGGGATTCAGCTTC |
| *Arg1* | CTCCAAGCCAAAGTCCTTAGAG | AGGAGCTGTCATTAGGGACATC |
| *IL4* | GGTCTCAACCCCCAGCTAGT | GCCGATGATCTCTCTCAAGTGAT |
| *Mmp9* | CTGGACAGCCAGACACTAAAG | CTCGCGGCAAGTCTTCAGAG |
| *β-actin* | CATCGTACTCCTGCTTGCTG | AGCGCAAGTACTCTGTGTGG |
